# Make My Day – Stroke Prevention Grounded in Engaging Everyday Activities in Primary Healthcare – A Single-Blinded Randomised Controlled Trial

**DOI:** 10.1177/21501319251385889

**Published:** 2025-11-02

**Authors:** Cecilia Johnsson, Eric Asaba, Susanne Guidetti, Elisabet Åkesson, Maria Hagströmer, Ann-Helen Patomella

**Affiliations:** 1 Department of Neurobiology, Care Sciences and Society, Division of Occupational Therapy, Karolinska Institutet, Stockholm, Sweden; 2 Unit of Research, Development, and Education, Stockholms Sjukhem Foundation, Sweden; 3 Department of Health and Rehabilitation, Sahlgrenska Academy, University of Gothenburg, Sweden; 4 Medical Unit Allied Health Professionals, Womens Health Theme, Karolinska University Hospital, Stockholm, Sweden; 5 Department of Neurobiology, Care Sciences and Society, Division of Neurogeriatrics, Karolinska Institutet, Stockholm, Sweden; 6 Academic Primary Health Care Centre, Region Stockholm, Sweden; 7 Department of Health Promoting Sciences, Sophiahemmet University, Stockholm, Sweden; 8 Department of Neurobiology, Care Sciences and Society, Division of Physiotherapy, Karolinska Institutet, Stockholm, Sweden

**Keywords:** engaging everyday activities, lifestyle habits, occupational science, primary healthcare, occupational therapy, stroke prevention intervention

## Abstract

Lifestyle habits significantly impact health, including stroke risk, and structured approaches in primary healthcare are needed to address risk factors and promote healthy lifestyles. Make My Day, a multifactorial activity-focussed and lifestyle-based stroke prevention programme, was developed to address the rising challenges of increasingly unhealthy lifestyle habits and stroke risk factors. This study evaluated the effect of the Make My Day intervention to reduce stroke risk at 12 months in primary healthcare. In this single-blinded randomised controlled trial, 122 individuals at risk for stroke were randomised to intervention group (n = 63) or control group (n = 59). Inferential statistics and a longitudinal mixed-effect logistic regression model were used to analyse the primary outcome. Inferential statistics were applied to analyse secondary outcomes, that is, goal achievement. At 12 months, the odds for high stroke risk (adjusted OR (CI) = 0.390 (0.156; 0.971), *P* = .043), was found significantly lower in the intervention group, compared to the control group. Both groups showed significant improvements in several stroke risk factors and goal fulfilment at 12 months. The Make My Day intervention trial indicates a reduction in stroke risk, and the potential of individual goals and engaging everyday activities to support lifestyle change. Make My Day can be a valuable means to address stroke prevention within primary healthcare.

The study is registered at ClinicalTrials.gov, Identifier: NCT05279508. Protocol ID: KI2020-00175.

## Introduction

Lifestyle habits are determinants of health and disease.^1,2^ Policies, healthcare guidelines and recommendations have shifted focus from mainly curative and reactive approaches to prevention and proactive measures to address the escalating global health issues associated with non-communicable diseases (NCDs).^2-4^ Globally, the prevalence of NCDs, including stroke, is high.^5,6^ Although the incidence has declined in Western nations, including Sweden,^1,7,8^ cardiovascular diseases (CVD), including stroke, continue to be the leading causes of mortality and disability.^9^ Notably, 80% of stroke cases are attributable to modifiable risk factors such as hypertension, insufficient physical activity, dietary patterns, obesity, and tobacco use.^10^ These modifiable risk factors are universally recognised as contributors to stroke, and addressing them presents significant opportunities for prevention and reducing stroke risk,^11^ yielding profound benefits for individuals and society. There is an urgent need for effective interventions promoting healthy lifestyle habits, targeting modifiable risk factors to reduce the risk of CVD.^12^ However, there is still a lack of consensus concerning how to support lifestyle-based stroke prevention to best mitigate risk factors and promote healthy lifestyle changes within primary healthcare settings.^13-15^

Previous studies on the prevention of stroke have predominantly focussed on a single risk factor, such as diet, or specific diagnoses, such as diabetes or obesity.^16,17^ However, multifactorial prevention interventions for stroke, especially interdisciplinary approaches, have been highly effective and have positive outcomes in addressing risk factors and promoting lifestyle changes.^18,19^ Engaging in health-promoting, meaningful everyday activities has been shown to positively impact everyday life and healthier lifestyles,^20-22^ but requires further exploration in prevention intervention research. An engaging everyday activity (EEA) is a highly meaningful activity that engages or exhilarates, provides a sense of intense participation, and is performed regularly.^23^ EEAs entail activities from all parts of a person’s life, that is, it could be a social, cultural, spiritual, or physical activity, performed together with others, in solitude or in a contextual community. What defines an EEA is that it is defined based on an individual experience, such as singing in a choir, socialising with friends, gardening, or reading a good book, and it can change for a person over time. However, depending on its nature and the context in which it is performed, an EEA can facilitate both health and ill-health^24^ such as in the latter case, socialising with friends while consuming large amounts of alcohol. There is insufficient knowledge regarding the effect of multifactorial prevention interventions aimed at stroke prevention and delivered through an interdisciplinary team. Furthermore, it is important to investigate how a prevention intervention within primary healthcare based on EEA can facilitate behaviour change, enhance sustainable lifestyle prevention, and ultimately contribute to reducing stroke risk.

Make My Day (MMD) is a multifactorial intervention grounded in EEA to promote healthy everyday lifestyle habits, addressing modifiable stroke risk factors and aimed to reduce stroke risk augmented by an app.^25^ The MMD intervention is distinguished from other interventions targeting lifestyle and behaviour change as it utilises self-defined and health-promoting EEA to facilitate sustainable lifestyle changes. With their facilitating possibilities for lifestyle change, EEAs are both the means and the goal for health and well-being in MMD. The MMD intervention is grounded in and builds on foundations in occupational science, models from occupational therapy, and behavioural change theory, that is EEA,^24^ person-centred goal setting,^26^ impact of self-monitoring and feedback on behaviour change.^27^ In accordance with the Medical Research Council’s guidelines for developing and evaluating complex interventions,^28,29^ pre-,^14^ feasibility,^30^ and pilot-^31^ studies have been conducted. The MMD intervention has shown good acceptability and is feasible to apply for the purpose of reducing stroke risk.^22,30,31^ The aim of this study was to evaluate the effect of the Make My Day intervention to reduce stroke risk in a primary healthcare setting.

The study hypothesis was that study participants, with an identified risk for stroke, randomised to the MMD intervention programme, were more likely to decrease their stroke risk compared to those randomised to the control group.

## Methods

### Trial Design

A single-blinded Randomised Control Trial (RCT) was designed and reported in accordance with the Consolidated Standards of Reporting Trials.^32^ The study is registered at ClinicalTrials.gov Identifier: NCT05279508. A study protocol describing the randomisation, set-up, and content of the MMD intervention programme has been previously published.^25^

### Study Setting

The study was situated in Swedish primary healthcare at 3 primary healthcare (PHC) rehabilitation units in different areas of Region Stockholm. Swedish PHC is funded by tax-based social insurance for all citizens, with no requirement for referrals. PHC clinics were recruited, striving for geographical diversity. Each PHC unit was required to have a rehabilitation team including an occupational therapist, physiotherapist, and dietitian, and accommodate a group of up to 16 study participants for the intervention programme. Additional criteria for the PHC included dedicating time for the MMD education, preparation, and intervention delivery. Prior to implementing the MMD intervention programme, healthcare professionals (HP) completed a preparatory education provided by the research team, which covered the background of the MMD intervention, how to operate the online learning platform, and how to conduct each session using the provided PowerPoint slide decks. More detailed information about the education has previously been described by Jakobsson et al.^33^

### Participant Recruitment and Eligibility Criteria

Participants were recruited at 3 time-points between 2022 and 2023 through social media, including Facebook, through the university media agency, local newspaper advertisements, and paper and digital flyers at primary healthcare clinics in Region XX. The registration was accessed through a link or QR code to a secure electronic platform, REDCap,^34^ which provided study information, a consent form, and a self-assessment of stroke risk and demographic data. Inclusion was based on assessed stroke risk using the stroke risk scorecard (Table 1).^35^ Persons with ≥3 high stroke risk factors, of which ≥2 were modifiable (ie, high blood pressure, overweight, low physical activity, and smoking) were included in the study. Additional inclusion criteria were (a) age 55 to 75 years, (b) living in the greater Stockholm region, c) motivation to lifestyle change, by answering a motivation to change survey, and (d) no cognitive impairment hindering participation. Exclusion criteria were (a) previous stroke or TIA diagnosis, (b) inability to use a smartphone application, and (c) lack of understanding of the Swedish language.

**Table 1.** Instruments Used in the MMD Intervention.

Outcome	Instrument	Purpose	Measure	Performed
**Primary outcome**				
**Stroke risk**	Stroke risk score card (SRSC)^35^	*Overall stroke risk combining modifiable and non-modifiable risk factors*	A checklist with 8 risk factor domains each with 3 levels (red, yellow, and green). The score, summing up each level provides the present overall stroke risk: *3 red* *=* *High Stroke Risk*, *4–6 yellow* *=* *Caution*, *6–8 green* *=* *Low Stroke Risk*	Onsite
**Secondary outcomes**				
**Stroke risk**	The Stroke Riskometer (SRM)^36^	*Long-term stroke risk prediction combining modifiable and non-modifiable risk factors*	Multiple questions on own health, physical measures, genetics, and lifestyle habits. An algorithm, validated by comparing predictions against 2 established stroke risk models, sums up the predicted absolute stroke risk in 5 years and in 10 years in % and a ratio compared to a person of the same gender, age, and ethnicity.	REDCap
**Activity performance and satisfaction**	Canadian Occupational Performance Measure (COPM)^26^	*Perceived performance and satisfaction with activities in everyday life.*	Scale ranging from 1 to 10 in 2 aspects: (i) current performance, *1* *=* *not able to perform the activity at all; 10* *=* *able to do it extremely well; and (ii) satisfaction with doing*, *1* *=* *not satisfied ;10* *=* *extremely satisfied.*	Onsite
**Quality of life**	EQ-Visual Analogue Scale (EQ-VAS)^37^	*Perceived state of health as a whole.*	Scale 0–100: *0* *=* *worst possible health*, *100* *=* *best possible health.*	REDCap
**Life satisfaction**	Life Satisfaction Scale11 (LiSat-11)^38^	*Perceived satisfaction with life “Satisfaction with life as a whole”*	Scale 1–6: *1* *=* *not satisfied to* *6* *=* *very satisfied*	REDCap
**Lifestyle habits**	Lifestyle habit survey^39^	*Self-reported lifestyle habits in everyday life: tobacco use, alcohol consumption, physical activity, and eating habits*	Four habits with 11 questions in total, subdivided into 4–5 levels of performance during a week or the last months. For example, “how often do you eat fruit or berries?” *1* *=* *2 times each day or more* *2* *=* *One’s a day* *3* *=* *A couple of times during a week* *4* *=* *One time, or less, during a week.*	REDCap
**Physical activity levels and patterns**	ActivPAL^®40^	*To objectively measure physical activity levels and patterns*	A small device attached by a waterproof dressing to the thigh. Measure activity levels and patterns during the whole day for 5 days. For example, how much time spent lying down, sedentary, standing, or walking and when during the day each pattern was performed	Onsite
**Anthropometric measures**	Body Mass Index (BMI)	*Indicator for body fat and weight status. Height and weight. Weight in kilograms (kg).*	<18.5 = Underweight, 18.5-24.9 = Normal, 25.0-29.9 = Overweight, >30.0 = Obesity	Onesite
	Blood pressure^41^	*Measurement of systolic and diastolic blood pressure*	High blood pressure: > 140/90, Elevated blood pressure: 120–139/80–89, Normal blood pressure: < 120/80. A decrease/increase of 10 mm Hg in systolic blood pressure and 5 mm Hg in diastolic blood pressure was considered a clinically meaningful change.	Onsite
	Waist circumference^8^	*Measure of waist circumference in centimetres (cm)*	Women: ≥88 cm – increased risk of type 2 diabetes, heart disease, high blood pressure, high cholesterol, and stroke Men: ≥102 cm – increased risk of type 2 diabetes, heart disease, high blood pressure, high cholesterol, and stroke	
**Participation in health-promoting activities**	Participation in health-promoting activities (PHPA) questionnaire	*Perceived participation in activities in everyday life that can increase or decrease health.*	10 statements with 5 levels of agreement. For example, “I participate in activities positive for my health,” “I participate in meaningful activities.” *1* *=* *No, do not participate*, *2* *=* *Sometimes (less than once a week)*, *3* *=* *Often (up to twice a week)*, *4* *=* *regularly (3 or more times a week)*, *5* *=* *Daily*	REDCap
**Perception of balance in everyday life**	Occupational Balance Questionnaire (OBQ)^42^	*Perception of balance within or between different occupations in everyday life.*	13 statements with 4 level of agreements: *1* *=* *Do not agree at all* *2* *=* *Agree partially* *3* *=* *Agree a lot* *4* *=* *Totally agree*, with a sum ranging from 0–39.	REDCap
**Health literary**	European Health Literacy Questionnaire (HLS-EU-Q16)^43^	*Questions indication health literacy and perceived ability to find, understand, and use health and diseases related information.*	16 questions related to ability and understanding with 4 levels ranging from *1* *=* *very easy* *2* *=* *easy* *3* *=* *difficult* *4* *=* *very difficult*	REDCap

All instruments were used at all 3 timepoints for both the intervention group and the control group.

**Figure 1.**
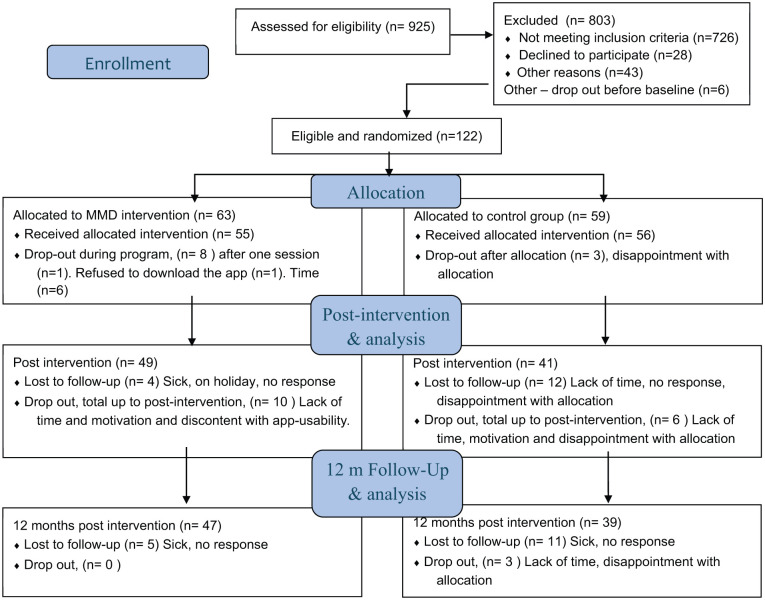
Participant inclusion flowchart following the CONSORT. The app = not willing to download the app, Time = lack of time. Total Dropout in the intervention, n = 10, and in the controls, n = 9. LTFU = Lost to follow-up.

After informed consent and an initial examination of the self-reported screening data, conducted by the first author, eligible persons were contacted via their preferred method of contact – email or phone – to arrange a recruitment interview. Three researchers conducted the recruitment interviews over the telephone following a programme manual. The programme manual was redeveloped by the research team based on results from the previous pilot study to align with the RCT protocol. Comprising instructions and timeframes for recruitment interviews, data collection, and the intervention programme, the manual guided and supported the researchers. The recruitment interview included follow-up questions regarding the above-stated inclusion and exclusion criteria. Moreover, it provided detailed information on conditions for participation, such as the possibility of being randomised either to the control group (CG) or the intervention group (IG) and the time needed for the intervention programme. The decision of inclusion or exclusion from the study was made following the recruitment interview, where non-eligible persons who did not meet the inclusion criteria or met exclusion criteria, did not want to accept the conditions of the study, or were unable to be reached, were excluded. Additionally, eligible persons declined participation due to time constraints or geographical issues. A total of 128 persons fulfilled the inclusion criteria. Six people later declined and therefore did not participate in the baseline measures. Hence, 122 persons participated in baseline measures and the following randomisation. (Figure 1)

### Ethical Considerations

during initial recruitment and again at baseline. The study was approved by the Swedish Ethical Review Authority, Sweden (Ref. numbers: 2015/834-31, 2016/2203-32, 2019/01444, and 2021-05902-02). Due to the sensitivity of some narratives shared by the participants, the data collectors and researchers had regular debriefing sessions and reflections on how to best support the participants. When untreated health issues, such as high blood pressure, were detected, the participant was strongly advised to seek medical attention at their local primary healthcare clinic. Moreover, if relevant, the participants were provided information about treatment programmes focussing on smoking cessation and/or alcohol consumption reduction.

### Data Collection

**Figure 2.**
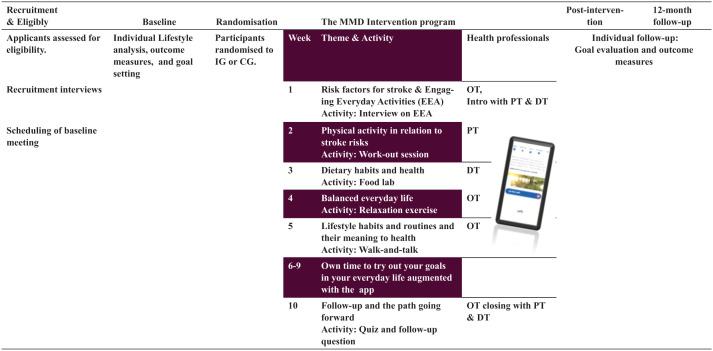
lllustrates the timeline of the MMD intervention and the content of the MMD intervention programme conducted by healthcare professionals, specifically an occupational therapist (OT), a physiotherapist (PT), and a dietitian (DT), and the MMD app augmenting the intervention. MMD (Make My Day). IG = Intervention group, CG = Control group.

Data collection took place from spring 2022 through spring 2024. There were 5 cohorts, with the first MMD intervention conducted in spring 2022 and the fifth and final intervention in spring 2023, each spanning over 12 months. Data collection for each cohort occurred at 3 time points: baseline, post-intervention at weeks 11 and 12 after the final MMD session in week 10, hereafter referred to as post-intervention, and 12-month follow-up (Figure 2). The researchers (n = 4) conducting data collection held calibration meetings before and during the data collection periods and kept reflexive notes. These calibration meetings and reflective notes also served as quality assurance, as it was not always the same researcher who initially met the participant at baseline who conducted the follow-up assessment. Additionally, the data-collecting researchers were blinded to allocation, and participants were asked not to disclose their allocation to the researchers. Data collection commenced when participants received an email containing a link to questionnaires (Table 1) on the REDCap platform to complete, which took approximately 15 to 20 min, followed by an individual meeting as described below.

### Baseline Assessment

At baseline, all participants had an individual meeting with a researcher. The MMD programme manual guided the data collection and comprised 3 parts at baseline: (a) a lifestyle analysis based on stroke risks from the Stroke risk scorecard (SRSC) and lifestyle habits, (b) anthropometric measures (blood pressure, weight, and waist circumference), and (c) the Canadian Occupational Performance Measure (COPM)^26^ to formulate 3 individual activity-focussed goals related to the lifestyle analysis chosen and prioritised by the participant. The COPM was developed to facilitate client-centred goal setting through an interview focussing on participation in everyday activities. In the interview, the researchers employed a motivational interview (MI) approach, meaning a collaborative, person-centred approach to guide towards change by facilitating exploration and identifying areas for change^44^ in this case, formulated as activity-focussed goals. The goals were rated using an activity performance scale (1-10) and satisfaction with the performance scale (1-10).^26^ In this study, COPM focussed on incorporating EEA in the goal setting, serving as a tool to measure the perceived performance and satisfaction of participation in EEA. Additionally, all participants were asked to wear an activity monitor for 5 days to measure physical activity levels and patterns. This comprehensive assessment, along with evaluation of the formulated goals, was reiterated with the participants at the 2 follow-up sessions scheduled at post-intervention and 12 months post-baseline.

### Randomisation

Following baseline assessment, a block randomisation 2 + 2 (2 controls = A and 2 interventions = B, with blocks of 4 having random block orders: AABB, ABAB, ABBA, BABA, BAAB, and BBAA) was performed to allocate participants to the intervention group (IG) or control group (CG). The allocation was performed by a researcher not involved in the data collection or intervention delivery.^25^

### The Make My Day Stroke Prevention Intervention Programme

In addition to the lifestyle analysis and formulated person-centred activity-focussed goals at baseline, the IG participated in the MMD intervention programme led by allied HP in PHC. This interprofessional team collaborated to facilitate a supportive environment for persons at risk for stroke to work towards enhancing a healthy lifestyle and achieving their goals. Over 10 weeks, participants attended 6 group sessions focussing on addressing stroke risk factors, EEA, and lifestyle changes. The promoted lifestyle habits encompass physical activity, healthy cooking, and eating habits in accordance with international^7,8^ and national guidelines for CVD and stroke prevention.^1^ Moreover, the participants had access to the MMD app to self-report goal fulfilment, lifestyle habits, and participation in EEA, as well as to an information library on the intervention content (see Figure 2). The intervention content is further described in detail elsewhere.^25^ EEAs are seen as key to change in MMD and, therefore, incorporated into every session of the intervention programme as a constructive alignment through the intervention. For example, in the session on physical activity, focussing on EEA means being active by doing activities that foster motivation and engagement, such as dancing, playing tennis with friends, or taking nature walks with a camera for photography.

### The Control Group

The CG received the same lifestyle analysis, formulated 3 person-centred, activity-focussed goals focussed on incorporating EEA with an MI approach as those in the IG, and attended the 2 follow-up assessments. Although the control group formulated goals incorporating activities that were important and meaningful to the person, the concept of EEA was not introduced at baseline. After the randomisation, the controls had the possibility to self-manage the goals set in the lifestyle analysis.

### Objectives and Outcomes

The primary objective was to examine overall stroke risk as an outcome with the SRSC, a checklist with 8 risk factor domains, each with 3 levels: high, moderate, or low risk. The score, summing up each level, provides the current overall stroke risk.^35^ To complement the overall present stroke risk provided by the SRSC, a secondary objective was to examine long-term stroke risk prediction over 5 and 10 years using the Stroke Riskometer (SRM).^37^ The SRM entails, in addition to the variables included in the SRSC, variables such as sex, age, ethnicity, different heart conditions, and cognitive functions. The SRM accumulates risk factors and calculates the predicted stroke risk over time. Additionally, a secondary objective was to examine factors affecting and affected by stroke risk using activity performance and satisfaction (COPM),^26^ quality of life (EQ-VAS),^38^ life satisfaction (LiSat),^39^ lifestyle habits,^40^ activity balance (OBQ),^41^ health literacy (HLS-EU-Q16),^42^ participation in health-promoting activities (PHPA), anthropometric outcomes, and objective levels and patterns of physical activity (ActivePAL)^43^ (see Table 1). The primary time-point of interest was 12 months, but also post-intervention scores were of interest to follow the process. The SRSC, which provides self-reported data, has not been sufficiently psychometrically tested, although it has been used in a few studies, including the MMD pilot study.^31^ All remaining outcome instruments are validated for a broader population. Taking less than 5000 steps/day has been proposed as a cut-point for an increased risk of ill-health and premature death in a similar population to that included in this.^45^ The outcomes and procedure are described in detail in a study protocol.^25^

## Research Questions

- What is the effect of the MMD intervention programme on the IG stroke risk compared to the CG, measured with the SRSC and SRM at post-intervention and 12 months?
- Has there been a change in lifestyle factors following participation in the MMD intervention programme in the IG compared to the CG, measured with the lifestyle habit survey, EQ-Vas, LiSat, OBQ, HLS-EU-Q16, PHAP, and ActivPAL, and if so, which specific factors have changed?
- What was the effect on perceived activity performance and satisfaction through goal fulfilment measured with COPM incorporating EEA after attending the MMD intervention programme in the IG compared to the CG, at post-intervention and 12 months?

### Sample Size and Power Calculations

The power calculation was performed using the webpage Sample Size Calculator.^46^ The primary outcome was stroke risk at 12 months using the SRSC and the probability of staying in the high-risk group. Assuming an incidence of 60% in the high-risk group for controls and 30% in the intervention at the end of the study, 42 participants in each group are needed to detect this difference in independent proportions with 80% statistical power at a significance level of 5%. The assumption was based on the pilot study results.^31^ Additionally, a 30% dropout rate was assumed, requiring an enrolment of 118 participants (59 in each group) in the study.^25^

### Data Analysis

The data was examined for outliers and normal distribution by visual inspection of boxplots, histograms, and Q-Q plots.

Analysis of the primary outcome measured with SRSC was performed in stages. Firstly, descriptive statistics were used on the 3 SRSC levels (low, moderate, and high risk) to describe shifts in the levels at all time points in both the IG and the CG separately. Secondly, the SRSC was dichotomised into high risk (HR) and moderate risk/low risk (MR/LR) for bivariate analysis using the Chi-square test. Lastly, to measure the effect of MMD on reducing stroke risk over time, a longitudinal mixed effects logistic regression model, a robust model accounting for covariates and a flexibility for missing data, with a bilateral stroke risk outcome (lowered overall stroke risk or not lowered) to estimate odds ratios (ORs) and CIs was used. High risk was the dependent variable, and group (IG/CG) was the independent variable. The model included covariates (ie, age, sex, physical activity, fruit and green consumption, antihypertensive drugs, BMI, group [IG and CG], and time [post-intervention and 12 months]) and considered an interaction term between time and group. Time was included as a categorical variable with 12 months being the reference category, showing the trend of the slope of reduced stroke risk over time.

Normally distributed variables were analysed between IG and CG with repeated measures analysis of variance (ANOVA), post-hoc comparison between groups, within groups, and over time. The estimated mean differences and their 95% confidence intervals (CI) are presented.

Non-symmetrically distributed data and ordinal variables were analysed between IG and CG at different time points with the Kruskal-Wallis test. Mann-Whitney *U* and Hodges-Lehmann Median differences tests were used to calculate the estimated effect of the MMD intervention on absolute and relative stroke risk at post-intervention and 12 months, respectively. The estimated differences in medians can be seen as a potentially relevant clinical effect. Within-group analysis for the IG and CG at baseline, at post-intervention, and at 12-month follow-up was analysed with the Related-samples Friedman’s test with post-hoc comparison.

Nominal variables and dichotomised data were analysed between IG and CG at different time points with the Chi-square test and within-groups at each time point with the Marginal Homogeneity test. Test for differences in proportions was performed on binomial outcome variables between groups, with the Independent samples proportions test, and within groups, with the paired-samples proportions test. Results are displayed with estimated proportions and their 95% CIs. Clinically meaningful changes are presented for: COPM ≥ 2p, and EQ-VAS ≥ 10p, that is, 2-point and 10-point increase from the compared value at baseline, respectively.

Two-sided *P*-values were reported, and the level of statistical significance was set at .05. The Bonferroni method was used to account for multiple comparisons at different time points. SPSS V.29.0.2^47^ and R version 4.4.1 (46) were used to analyse the data.

## Results

### Participants

Figure 1 shows the flowchart for the 122 participants included and randomised to IG (n = 63) or to the CG (n = 59). The dropout rate in the IG was 15.9%, and in the CG, 15.2%. No systematic attrition was identified when comparing characteristics between completers and dropouts (Appendix 1). The main reasons were in the IG, a lack of time (70%), and in the CG, a lack of time and disappointment in allocation to the CG (44.4%). Lost to follow-up (LTFU) was post-intervention 7.7% in the IG and 20% in the CG, and at 12 months, 9.4% in the IG and 20.8% in the CG. No statistically significant differences were detected between the groups’ demographics and stroke risk characteristics at baseline (Table 2).

**Table 2.** Demographics and Stroke Risk Characteristics of the Study Participants at Baseline.

	Variables	Intervention group n = 63	Control group n = 59
Demographics	Age, *m* (SD)	61.7 (4.9)	63.5 (5.5)
	Sex (women) n (%)	31 (49.2)	30 (50.8)
	Basic education (grundskola) n (%)	13 (20.7)	11 (18.7)
	Higher education/university n (%)	50 (79.4)	48 (81.4)
	Country of birth, Sweden n (%)	38 (60)	46 (77)
	Ethnicity, Caucasian European n (%)	62 (98.4)	59 (100)
	Living with a partner n (%)	39 (62)	37 (63)
	Retired n (%)	3 (5)	4 (7)
	Yearly income, euro *35* *500-53* *200 € (40* *000-599* *000 SEK)* n (%)	46 (73.1)	41 (69.5)
	Very comfortable using a smartphone app n (%)	33 (52.4)	31 (52.5)
Stroke risk characteristics’	Overall high stroke risk (stroke risk score card) n (%)	63 (100)	59 (100)
	Systolic blood pressure, *m* (SD)	140.3 (14.2)	144.4 (20.3)
	Diastolic blood pressure, *m* (SD)	92.2 (9.2)	92.7 (11.1)
	Self-reported Stress and/or depression, Yes n (%)	34 (54)	22 (37.3)
	Self-reported Parents with stroke diagnosis, Yes n (%)	21 (33.3)	23 (39)
	Self-reported Diabetes type I or II, Yes n (%)	12 (19)	11 (18.6)
	Self-reported Heart disease, Yes n (%)	9 (14.3)	13 (22)
	Self-reported Arrythmia, Yes n (%)	10 (15.9)	9 (15.3)
	Weight (kg), *M* (IQR)	95.7 (16.4)	96.7 (23.8)
	Overweight, *BMI ≥25* n (%) Obesity, *BMI ≥ 30* n (%)	63 (100) 44 (69.8)	58 (98) 40 (67.8)
	BMI: *M* (IQR), min/max	32 (5.6), 26.1/43.9	31.5 (5.3), 24.2/46.9
	Waist circumference (cm), *M* (IQR)	112 (11.5)	110 (19)
	Self-reported physical activity (Not fulfilling recommended level of physical activity*) n (%)	37 (58)	26 (44)
	Self-reported dietary habits: risk/potential risk/healthy^1^ n (%)	14 (22)/34 (54)/15 (23)	13 (22)/38 (64.4)/8 (13.6)
	Self-reported smoking (Yes) n (%)	2 (3.7)	2 (3.4)
	Self-reported risk use of alcohol^1^ (Yes) n (%)	4 (6.3)	2 (3.4)

Demographics were collected through a demographic questionnaire, and the stroke risk characteristics are risk factors from the Stroke Riskometer, the Lifestyle survey questionnaire, and measures performed at baseline.

Abbreviations: *m* (SD), mean (standard deviation); *M* (IQR), median (interquartile range. Min/Max = minimum/maximum); kg, kilograms; BMI, Body Mass Index; cm, centimetres.

* World Health Organisation (WHO) guidelines on physical activity and sedentary behaviour 2020.

1 National Guidelines for prevention and treatment of unhealthy lifestyle habits.

### Primary Outcome

#### Overall Stroke Risk, Stroke Risk Score Card

**Figure 3.**
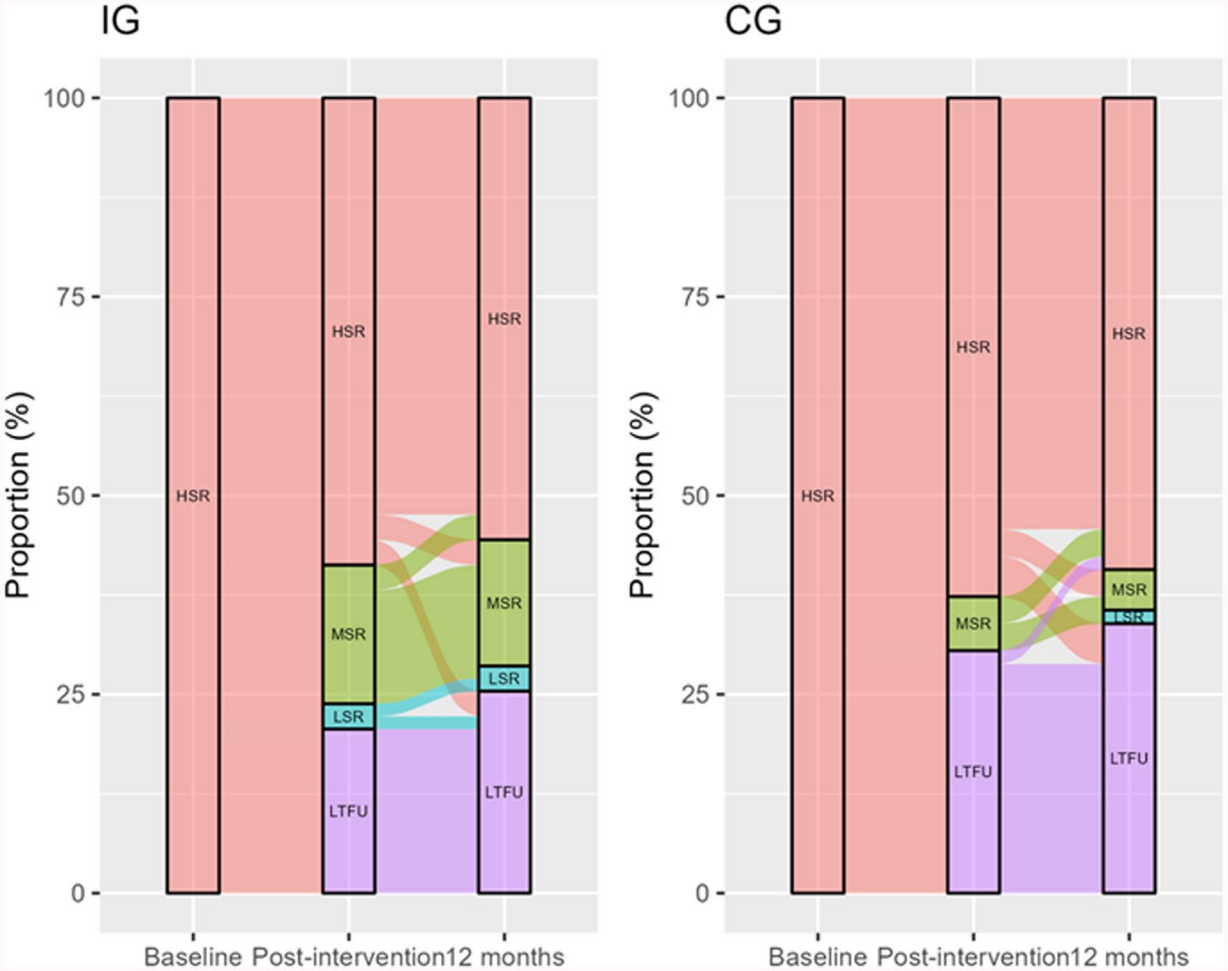
Alluvial plot depicting the shift in stroke risk measured with the SRSC and LTFU over time. The flow represents transitions between the levels of stroke risk from baseline to post-intervention and 12-month follow-up among participants in IG and CG, respectively. The colours indicate different levels of stroke risk and LTFU, and the width of the flows corresponds to the proportion of participants transitioning from 1 stroke risk to another. IG = Intervention group, CG = Control group. SRSC = stroke risk scorecard. HSR = high stroke risk, MSR = moderate stroke risk, LSR = low stroke risk, LTFU = lost to follow-up.

All participants had a high overall stroke risk at inclusion, assessed with SRSC (Table 2). In the longitudinal multivariable mixed effects logistic regression analysis, an adjusted OR (CI) of 0.390 (0.156; 0.971, *P* = .043) at 12 months and all other variables at the reference indicated that allocation to the IG significantly lowered the odds for high stroke risk over time. The model, taking time dependence over time into account, was a good fit with 82% prediction accuracy. No statistically significant interaction between time and group was identified. Hence, the interaction term was excluded from the final model. No statistically significant differences were found for the other included variables (age, sex, physical activity, fruit and green consumption, use of antihypertensive drugs, BMI, and time). The Chi-square test on stroke reduction, which does not take dependence over time or adjustments to covariates into account, showed no significant difference at 12 months between groups. However, post-intervention, a significant stroke risk reduction was detected (Chi-Square = 3.913, df = 1, *P* = .048) in the IG compared to the CG. Post-intervention 22% in the IG lowered their stroke risk 1 step from high to moderate, and 4% lowered their stroke risk 2 steps from high to low risk compared to baseline. In the CG, 10% lowered their stroke risk, 1 step from high to moderate risk, while no one lowered their stroke risk 2 steps from high to low risk. At 12-month follow-up, the IG maintained its lowered risk, with 21% having lowered the risk to moderate risk and 4% to low risk. The CG had further reduced its risk with 8% lowering the high risk to moderate risk, and 3% to low risk (see Figure 3).

### Secondary Outcomes

#### Long-Term Stroke Risk Prediction

At the 12-month follow-up the MMD intervention programme and the output of the SRM, an estimated median difference (95% CI) of 1.681 (–0.66; 4.05) indicated a potentially relevant small clinical effect on the 10-year absolute stroke risk prediction. No effect was detected on the 5-year absolute stroke risk or 5- and 10-year relative stroke risk at any time point.

#### Activity Performance and Satisfaction

No significant differences in perceived activity performance or satisfaction through goal fulfilment, measured with COPM on the mean score, were observed at any time point between the IG and the CG. However, a significant difference in reaching a clinically meaningful change, that is, a change of ≥2p, in activity performance through goal fulfilment for the IG was detected post-intervention (*P* = .015). No significant difference was detected at 12 months. (Table 3).

**Table 3.** Activity Performance and Satisfaction Measured with COPM at Post-intervention and 12 months.

COPM	Activity performance post-intervention n = 92			Activity satisfaction post-intervention			Activity performance 12m n = 86			Activity satisfaction 12m		
	IG n = 47	CG n = 44	P (CI)	IG n = 47	CG n = 44	P (CI)	IG n = 46	CG n = 40	P (CI)	IG n = 44	CG n = 40	P (CI)
≥2p, n (%)	36(76.6)	23(52.3)	0.015 (.05; .42)	35(74.5)	26(59.1)	0.119 (−.04; .33)	35(76.1)	26(65)	0.259 (−.08; .30)	35(76.1)	23(57.5)	0.067 (−.02; .37)

Between group comparisons with Independent-sample proportions test *P* = 2-sided significance (Wald H0), *P* < .05, Confidence Interval (CI) 95%. Cut off at ≥ 2p = Clinically meaningful change compared to baseline measures.

Abbreviations: IG, Intervention group; CG, Control group; COPM, Canadian Occupational Performance Measure.

#### Lifestyle Factors and Habits

Table 4 describes between-group comparisons of lifestyle factors and lifestyle habits at all time points. A significantly larger fruit and greens consumption, *P* = .017 (0.14; 0.78), and participation in health-promoting activities *P* = .012 (–0.16; 0.48), was detected in the IG compared to the CG post-intervention. At the 12-month follow-up, the IG reported to perceive more symptoms of stress and depression, *P* = .039 (0.01; 0.34), compared to the CG. There were no significant differences between IG and CG in any other lifestyle-related variables such as self-reported and objective physical activity, intake of fruits and greens, participation in health-promoting activities, quality of life, lifestyle satisfaction, and systolic or diastolic blood pressure at any time-point.

**Table 4.** Between-Group Comparisons of Lifestyle Factors.

Health measurements		Baseline IG	Baseline CG	*P*-value/ Estimate CI (95%)	Post-intervention IG	Post-intervention CG	*P*-value/ Estimate CI (95%)	12 months IG	12 months CG	*P*-value/ Estimate CI (95%)
**Lifestyle factors**	Systolic blood pressure *m* (SD) ^a^	140.3 (14.2)	144.3 (20.3)	.199 (–10.3; 2.2)	137.1 (15.9)	142 (19.2)	.200 (–12.4; 2.6)	136.7 (17.7)	136 (17.4)	.866 (–6.9; 8)
	Diastolic blood pressure *m* (SD) ^a^	92.3 (9.1)	92.7 (11.2)	.802 (–4.1; 3.1)	88.6 (9.4)	89.3 (11.3)	.758 (–5.1; 3.7)	87.7 (9.8)	84.9 (9.5)	.198 (–1.5; 6.9)
	Weight kg *M* (IQR) ^b^	94.2 (17.6)	96.7 (24.3)	.975 ( –6.5; 5.8)	93 (17)	95.8 (23)	.782 (–5.9; 6.9)	93.5 (18.8)	95.2 (25.6)	.735 (–7.8; 5.9)
	BMI *M* (IQR) ^b^	32 (5.6)	31.5 (5.3)	.360 (–1.9; .8)	31.4 (4.9)	31 (5.2)	.276 (–2.5; .73)	30.9 (4.5)	30.7 (4.3)	.288 (–2.9; .61)
**Lifestyle habits**	Number of fruit and greens /day *M* (IQR) ^c^	2 (1)	2 (1)	.32 (−.09; .54)	2 (1)	2 (1)	.017 (.14; .78)	2 (1)	2 (1)	.89 (−.44; .43)
	Physical activity level *m* (IQR) ^c^	2 (1)	2 (2)	.77 (−.34; .26)	2 (1)	2 (1)	.63 (−.26; .42)	2 (2)	2 (2)	.55 (−.49; .21)
	Self-reported stress or depression (yes) n (%) ^d^	34 (54)	22 (37)	.065 (−.01;.33)	23 (46)	15 (34.9)	.277 (−.09; .3)	18 (38.3)	7 (17.9)	.039 (.01; .34)
	Self-reported physical activity (yes) n% ^d^	26 (41.3)	33 (55.9)	.105 (−.32; .03)	35(74.5)	32 (72.7)	.851 (−.16; .19)	34 (66.7)	30 (58.8)	.413 (−.11; .26)
	Self-reported risk use Alcohol (yes) n (%) ^d^	4 (6.3)	2 (3.4)	.45 (−.06; .11)	3 (6.4)	1 (2.4)	.36 (−.06; .13)	2 (4.3)	1 (2.6)	.67 (−.08; .11)
	Self-reported smoking (yes) n (%) ^c^	2 (3.2)	2 (3.4)	.95 (−.08; .07)	1 (2)	2 (4.7)	.47 (−.12; .06)	1 (2.1)	2 (5.1)	.45 (−.13; .06)
	Participation in health-promoting activities m(IQR) ^c^	2 (1)	2 (1)	.55 (−.39; .13)	3 (1)	3 (2)	.012 (−.16; .48)	3 (1)	3 (2)	.83 (−.15; .47)
	Participation in meaningful activities m(IQR) ^c^	3 (2)	3 (2)	.39 (−.33; .20)	3 (1)	3 (1)	.40 (−.24; .40)	4 (1)	3 (1)	.078 (−.27; .37)
	Objectively measured daily physical activity >5000 steps/day n (%)d	49 (83.1)	44 (86.3)	.641 (−.17; .11)	33 (91.7)	28 (80)	.158 (−.05; .28)	24 (82.8)	29 (93.5)	.193 (−.27; .07)

Between group comparisons with ^a^ ANOVA *P* = .05 Confidence Interval (CI) 95%.

b Kruskal-Wallis test (CI from independent-samples Hodges-Lehman median differences).

c Chi-square test *P* < .05 (CI from Gamma) In R, ^d^%, Independent-samples proportions test Wald H0 *P* < .05 CI95%. Data on lifestyle factors were measured at the meeting with the researcher. Data on lifestyle habits were retrieved from the Lifestyle Habit Questionnaire, the Participation in Health Promoting Activities questionnaire, the Stroke Riskometer, and ActivPAL^®^.

Abbreviations: IG, Intervention group; CG, Control group; BMI, Body Mass Index; *m* (SD), mean (Standard deviation); *M* (IQR), median (interquartile range); n, numbers; kg, kilograms; cm, centimetres.

#### Within-Group Comparisons

As shown in Appendix 2, there was a statistically significant difference within-group reduction of high stroke risk factors and several modifiable risk factors in both the IG and the CG, that is, an improved perception of quality of life, an increase in self-reported physical activity level, an increase of activity balance, an increase of participation in meaningful activities, a reduction of waist circumference and a lowering of systolic and diastolic blood pressure. Additionally, a significant improvement in perceived activity performance, as measured by goal fulfilment, was observed in both groups at both time points.

## Discussion

This study evaluated the effect of the MMD stroke prevention intervention programme to reduce stroke risk. At 12 months, the longitudinal logistic regression analysis, adjusting for time and other covariates, detected a significantly lower odds for high stroke risk for the IG. However, the unadjusted test for group comparison showed no significant group difference. Post-intervention, a significant difference in reaching a clinically meaningful change in activity performance through goal fulfilment was detected in the IG compared to the CG. Both groups showed significant improvements in goal fulfilment and perceived activity performance, both post-intervention and at 12 months. A potentially relevant small clinical effect was indicated in the predicted 10-year absolute stroke risk at 12 months. These results show the potential of the MMD intervention to facilitate the reduction of high stroke risk over time, potentially leading to long-term, life-saving benefits.

Both IG and CG showed significant within-group changes in stroke risk factors at 12 months. This is, for example, highlighted by a shift at 12 months in the mean diastolic blood pressure with a reduction by >5 mmHg to 84.9 in the CG and 87.7 mmHg in the IG. A reduction by 5 mmHg in diastolic pressure has been assumed to reduce the risk of a cardiovascular event by 20% to 50% and mortality by 20%.^46^ The CGs’ results at 12 months are very interesting, indicating that participation in a research study per se has a positive outcome. This may be due to both groups performing a lifestyle analysis and formulating person-centred goals based on MI, and receiving post-intervention follow-up. MI has shown potential for lifestyle changes, where goal setting is identified as an important element,^36^ and in this study it seems to enhance results for both groups. Furthermore, at 12 months, the CG showed changes in stroke risk factors not observed in the IG, with a significant reduction in systolic blood pressure and weight. Increased awareness and literacy when meeting the research team repeatedly may also have empowered participants to seek medical assistance regarding that is, untreated high blood pressure.

Given that both the IG and CG received some level of intervention and demonstrated significant improvements, it is relevant to consider which specific intervention components are most effective. The detected significant difference in overall stroke risk may reflect the influence of group dynamics, person-centred realistic goals, and health literacy from the MMD intervention programme on the IG’s initial change process. One key component for the IG was the group setting, fostering reflections and discussions on various topics, including EEA. Another identified key component involved setting individual goals perceived as meaningful and encouraging.^48^ Earlier studies have shown that setting specific, relevant, and motivating goals in face-to-face meetings^49^ with active participant involvement,^21^ effectively promotes behaviour and lifestyle changes. As both the IG and CG showed significant improvement in goal fulfilment and perceived activity performance, incorporating EEA, realistic goals may have influenced the CG change process and reduced stroke risk. Although the authors expected that forming individual goals would also influence the CG, it turned out to be a powerful component for the change process. Through the lifestyle analysis and goal setting based on MI, the CG can be seen as an active CG.^50^ This shows the importance and promise of providing valuable means of motivating change to a high-risk group allocated to CG. None of the multifactorial lifestyle intervention studies in the review by Sisti et al^19^ and only one of the interprofessional lifestyle interventions highlighted in the review by Tapsell et al,^51^ mentioned active goal-setting with participants. In 1 study, the intervention group showed significantly better goal fulfilment than the controls, as goals were established post-MI, unlike the controls, who received predetermined goals.^52^ In contrast, MMD incorporates goal formulation and evaluation as behaviour change techniques,^27^ allowing both IG and CG to formulate their goals. This might explain the significant improvements in stroke risk factors detected in both the IG and CG, where the power of individualised activity-focussed goals incorporating EEA in facilitating lifestyle changes could have been underestimated. This underscores the effectiveness of tailored, activity-focussed goals in facilitating lifestyle changes. It also implies that a deeper understanding of EEA and its role in facilitating change can be a valuable means for HP in promoting lifestyle changes over time.

The pursuit of improving care through personalisation, collaboration, and preventive initiatives aligns the study results with the mandate given to PHC nationally^4^ and globally.^2,3^ However, prevention research shows that adherence to recommended guidelines for CVD and risk factor prevention remains low, highlighting a gap in the implementation of prevention strategies, identifying a need for more comprehensive preventive interventions that address lifestyle and risk factors holistically.^13^ The lack of established routines, tools, and organisational structure restricts PHCs’ capacity to help individuals shift their occupational habits towards healthier activities that can lower stroke risk.^14,53^ Goal setting, integrating EEA into everyday lives, is a promising, accessible, and acceptable means for PHC in their preventive efforts to promote health and prevent ill-health. MMD can be a valuable resource, systematically supporting preventive health practices and bridging gaps between PHC professionals in their preventive efforts. It highlights the capacity of allied HP in the prevention puzzle alongside general practitioners and nurses, dispersing the growing responsibility placed on primary healthcare, and potentially reducing future burdens and strain. However, this is a small-scale study within a given country context, and further research is needed in other healthcare and socioeconomic contexts.

### Methodological Considerations

A strength of this study is its longitudinal design, which follows participants for 12 months. It would have been even more valuable to have a 24- or 36-month follow-up to assess the sustainability of the lifestyle changes made. Although the perceived stress levels decreased for both groups, at 12 months, the IG reported higher stress than the CG. The IG showed significant improvements in quality of life and activity balance, unlike the CG. This suggests that while the MMD programme helped with planning everyday activities, it increased perceived stress in the IG. Stress awareness and stress-reducing activity could be a component to add to the intervention programme in future. The result might be due to being in the IG and wanting to succeed in performing lifestyle changes for themselves and for study compliance. The self-reporting of antihypertensive drug prescription by the participants does not give the full story, as this study did not oversee or comply with medical records. Moreover, participants might not be on antihypertensive drugs due to not having hypertension, not seeking healthcare, or due to suboptimal primary healthcare management. The IG and the CG received a lifestyle analysis and formulated activity-based lifestyle goals based on MI, which seems to be an intervention within the intervention, and the CG thereby became an active CG. Not distinctly separating the intervention formats may have affected the results, diminishing the detected significant differences.^50^ This is something to consider when assessing the results and planning for future studies. The SRM is used internationally both as a prevention strategy for lifestyle change^54^ and to assess stroke risk,^55^ though it is a novel approach as an outcome in an RCT study. There are, however, other equations for stroke risk calculation,^56^ which are under re-evaluation.^8^

Dropout in the IG was mainly caused by time constraints, while those in the CG cited both time constraints and dissatisfaction with allocation. This aligns with studies showing dropout due to disappointment with CG allocation, as participants expected to be in the IG.^57^ These issues highlight primary prevention research challenges, as all participants understood the programme’s structure, time commitment, allocation process, and consented at baseline. Dropout can be indicative of the intervention’s relevance on individual and societal levels, with participants eager to engage but underestimating the time required and the risk of being allocated to CG. Both IG and CG had LTFU, with higher rates in CG. Reasons are unknown, but low motivation from disappointment or lifestyle change challenges could be reasons. Prevention research indicates that many individuals struggle to maintain the lifestyle changes they have made over time due to diverse reasons, such as disruptions in their everyday life causing a drop in motivation, and reverting to their old habits.^58^ This highlights the challenges associated with maintaining motivation and sustainability in prevention interventions. A lesson learned and considerations for future intervention studies are the risk of a larger drop-out rate within the CG than the IG. The number of dropouts and LTFUs in this study caused a loss of power, which is a challenge in relation to feasibility. To address methodological limitations in the study, providing the MMD programme for the CG after completing the 12-month follow-up, ensuring equitable opportunity, possibly increasing motivation and reducing LTFU and drop-out rates.

The randomisation and blinding of data-collecting researchers are strengths. To further minimise the risk of bias, the researchers adhered to the programme manual, which provided detailed instructions for the measurements, and conducted trial runs and calibrations on each other prior to meeting the participants. Measurements such as blood pressure, waist circumference, and goal setting were performed on-site by the researchers and could be affected by assessor bias. To a large extent, the same researcher performed the assessments at each time point to reduce interrater variability. The recruitment process faced complexities due to a homogeneous sample of individuals with high education and income. Despite efforts, reaching vulnerable populations in socio-economically disadvantaged areas was challenging. Engaging these groups is a recognised research challenge, but strategies to improve accessibility and support can enhance participation.^59^ Two recruitment phases were implemented, with the second employing tailored social media advertisements to targeting underrepresented groups, socioeconomic areas, and males, which was successful. Flyers and pamphlets in clinics and newspapers yielded no applicants, but social media attracted resourceful applicants. Other potential causes for the homogeneous sample may include the inclusion criterion of being Swedish-speaking or the time required to devote to the intervention without financial compensation. To reach vulnerable populations, different recruitment strategies are needed. One possible approach may involve incorporating public health initiatives with multi-factorial approaches,^60^ such as public education strategies addressing lifestyle habits, their impact on health, and what society and individuals can do to promote health and well-being.

## Conclusion

At 12 months, a significant reduction in odds for high stroke risk was found in the intervention group when adjusting for covariates in a longitudinal analysis. The results suggest that the Make My Day (MMD) intervention may have positive effects on stroke risk reduction. Both intervention and control groups received a comprehensive lifestyle analysis based on motivational interviewing and formulated individual lifestyle goals incorporating participation in health-promoting engaging everyday activities. At 12 months, both groups demonstrated positive improvements in diastolic blood pressure, waist circumference, self-reported physical activity, and perceived activity performance through goal fulfilment. These results indicate the potential of the MMD intervention for primary healthcare settings and the potential of a comprehensive lifestyle analysis and formulating realistic individual goals.

## Appendix

**Appendix 1.** Attriation; LTFU Characteristics at Baseline.

Variables	Whole group n = 122	IG LTFU n = 6 (9.8%)	CG LTFU n = 13 (22%)
Gender W n (%) ^a^	61 (50)	4 (66.7)	11 (84.6)
Age m(SD) ^a^	62.6 (5.3)	62.3 (5.7)	59.5 (4.1)
Social Sbo n (%) ^b^	76 (62.3)	5 (83.3)	9 (69.2)
Education level, gymnasial n (%) ^b^	19 (15.6)	1 (16.7)	2 (15.4)
Yearly income, 200 000-399 000 n (%) ^b^	27 (22.1)	2 (33.3)	3 (23.1)
Profession white collar n (%) ^b^	84 (68.9)	4 (66.6)	10 (76.9)
Pensioner n (%) b	7 (5.7)	0 (0)	1 (7.7)
Sick leave last 12 month, yes n (%) ^b^	20 (16.4)	1 (16.7)	1 (7.7)
Weight *m* (SD) ^a^	97.7 (16.1)	91.9 (14.7)	91.9 (16.1)
Physical activity, not reaching recommended level n (%) ^c^	63 (51.6)	3 (50)	7 (53.8)
Unhealthy eating habits n (%) ^c^	27 (22.1)	1 (16.7)	3 (23.1)
Healthy eating habits n (%) ^c^	23 (18.9)	1 (16.7)	1 (7.7)
Systolic bp m(SD) ^a^	142.3 (17.5)	152 (12.5)	136.6 (16.5)
Self-assessment of stroke risk, 1-10 m(SD) ^b^	6.3 (1.6)	5 (2.3)	6.6 (1.2)
Motivation to change moderate n (%) ^d^	17 (14)	1 (16.7)	1 (7.7)
Multiple illnesses ^b^	23 (18.8)	2 (33.4)	4 (30.8)
Self-assessed overall health, 1-100 *m* (SD) ^b^	63.5 (17)	54.5 (22.6)	60.4 (16.2)
Risk use tobacco n (%) ^c^	4 (3.3)	1 (16.7)	0 (0)
Risk use alcohol n (%) ^c^	6 (4.9)	0 (0)	0 (0)
Health literacy index (problematic/inadequate) n (%) ^e^	24 (19.8)	0 (0)	1 (7.7)
Life as a whole, not satisfied n (%) ^f^	60 (49.2)	3 (50)	6 (46.2)
Given reason for LTFU ^g^			
Lack of time	NA	1 (16.7)	1 (7.7)
Lack of motivation	NA	1(16.7)	0
Sick/ill	NA	1(16.7)	3 (23.1)
Holliday	NA	2 (40)	0
No message	NA	1 (16.7)	9 (69.2)

Calculated with descriptive statistics on n (%) from the total n = 122.

a Stroke riskometer.

b self-reported at baseline assessment.

c Lifestyle habit survey.

d Motivation to change scale (5 questions on a scale 1-5 their motivation to change, a total score of 25, the higher the score the higher the motivation) 1

e European Health Litteracy Questionnaire (HLS-EU-Q16) dichotomised

f Life Satisfaction Scale11 (LiSat-11) dichotomised

g REDCap data registered by the data collectors.

Abbreviations: IG, Intervention group; CG, Control group; LTFU, lost to follow-up; *m* (SD), mean (standard deviation).

**Table:** Drop out numbers and reported cause.

Variables	Dropout IG n = 10 (16.3%)	Dropout CG n = 9 (15.2%)
Drop out period, n (%)	8 (80)	3 (33.3)
Gender w n (%)	5 (50)	5 (55.6)
Lack of time n (%)	7 (70)	4 (44.4)
Lack of motivation n (%)	1 (10)	4 (44.4)
Illness n (%)	2 (20)	0 (0%)
Family related n (%)	0	1 (11.1)
Other n (%)	0	0

Calculated with descriptive statistics on n (%) from the total n = 122. Data collected from REDCap registered by the data collectors.

Abbreviations: IG, Intervention group; CG, Control group.

**Appendix 2.** Within-Group Comparisons of Lifestyle Factors and Goal Fulfilment.

	Health measurement	Intervention		Control	
		Baseline – post-intervention *P*-value CI (95%)	Baseline – 12-months *P*-value CI (95%)	Baseline – post-intervention *P*-value CI (95%)	Baseline – 12-months *P*-value CI (95%)
**Lifestyle factors**	EQ – VAS (0-100) *M* (IQR) ^a^	0.005	0.002	0.095	0.126
	Systolic blood pressure *m* (SD) ^d^	0.100 (–0.45; 7.2)	0.061 (–0.15; 9.1)	0.120 (–0.91; 11.2)	0.004 (3.1; 19.8)
	Diastolic blood pressure *m* (SD) ^d^	0.002 (1.4; 7.5)	<0.001 (2.5; 8.3)	0.005 (1.1; 7.2)	<0.001 (3.7; 12.5)
	Weight (kg) ^a^	0.113	0.329	0.439	0.032
	BMI, *M* (IQR) ^a^	0.099	0.264	0.350	0.027
	Waist circumference (cm), *M* (IQR) ^a^	0.015	0.003	0.050	<0.001
**Lifestyle habits**	Number of fruit and greens /day *M* (IQR) ^b^	0.016	0.134	0.827	0.251
	Self-reported risk use Alcohol (yes) ^c^	0.20	0.163	0.763	0.285
	Physical activity level ^b^	<0.001	0.014	0.005	0.028
	Self-reported physical activity reaching recommended level (yes) ^c^	<0.001 (–0.45; –0.15)	0.007 (–0.42; –0.07)	0.020 (–0.29; –0.02)	0.631 –0.19; 0.12)
	Participation in health-promoting activities *M* (IQR) ^b^	<0.001	<0.001	0.026	0.245
	Participation in meaningful activities *M* (IQR) ^b^	0.076	0.018	0.691	0.392
	Self-reported Occupational balance *M* (IQR) ^a^	0.004	0.007	0.244	0.244
**Goal fulfilment**	COPM – perceived activity performance measured through goal fulfilment *M* (IQR) ^e^	<0.001 (–3.7; –2.7)	<0.001 (–3.8; –2.7)	<0.001 (–2.8; –1.3)	<0.001 (–3.3; –1.3)

Within group comparisons

a Friedmans test on the median (adjusted by the Bonferroni correction for multiple tests.

b Marginal Homogeneity test.

c Paired-samples proportions test (mid-*P* adjusted binomial). *P* < .05, 95% CI.

d RMANOVA within-subjects effects adjusted with Greenhouse-Geisser adjusted for multiple comparisons with Bonferroni (Bt).

e Wilcoxon, test, location parameter is given by the Hodges-Lehmann estimate.

Abbreviations: EQ – Vas, EQ Visual Analogue Scale; BMI, Body Mass Index; COPM, Canadian Occupational Performance Measure; *M* (IQR), median (interquartile range); kg, kilograms; cm, centimeters.
